# Supplementary material for: Mechanisms governing the pioneering and redistribution capabilities of the non-classical pioneer PU.1
Source: Nat Commun. 2020 Jan 21;11:402. doi: 10.1038/s41467-019-13960-2 (PMC6972792; doi:10.1038/s41467-019-13960-2)
Supplement: Supplementary file 7 — Source data [file 41467_2019_13960_MOESM7_ESM.zip › Source_Data/FigureS5/FigureS5B_MotifsETS1specificPeaks/homerResults/motif19.similar.html]

motif19

## Information for motif19

A
C
T
G
A
G
T
C
A
G
T
C
A
C
T
G
A
C
T
G
A
C
T
G
C
G
T
A
  
Reverse Opposite:  

A
C
G
T
A
G
T
C
A
G
T
C
A
G
T
C
C
T
A
G
A
C
T
G
A
G
T
C
  

|  |  |
| --- | --- |
| p-value: | 1e-17 |
| log p-value: | -4.049e+01 |
| Information Content per bp: | 1.950 |
| Number of Target Sequences with motif | 234.0 |
| Percentage of Target Sequences with motif | 20.62% |
| Number of Background Sequences with motif | 897.0 |
| Percentage of Background Sequences with motif | 10.99% |
| Average Position of motif in Targets | 101.4 +/- 53.9bp |
| Average Position of motif in Background | 95.9 +/- 77.4bp |
| Strand Bias (log2 ratio + to - strand density) | -0.2 |
| Multiplicity (# of sites on avg that occur together) | 1.10 |
| Motif File: | file (matrix) reverse opposite |

### Similar de novo motifs found

|  |  |  |  |  |  |  |  |
| --- | --- | --- | --- | --- | --- | --- | --- |
| Rank | Match Score | Redundant Motif | P-value | log P-value | % of Targets | % of Background | Motif file |
| 1 | 0.953 | A G C T A C T G A G T C A G T C A C T G A C T G A C T G C G T A | 1e-13 | -31.714451 | 9.60% | 3.95% | motif file (matrix) |
